# Effect of Occupation, on the Face

**Published:** 1889-07

**Authors:** 


### EFFECT OF OCCUPATION ON THE FACE.

A man's occupation or condition has a good deal to do with his facial expression. Intellectual pursuits, like studies of the scholarly professions, when coupled with temperate and moral habits of life, brighten the face and give a person a superior look. Magnanimity of nature or love of studies and arts will make a bright, glad face, but contrary to this a man may have a face that does not please anybody because of a love of self to the exclusion of all others, notwithstanding his learning and worldly shrewdness. Soldiers get a hard, severe look, overworked laborers constantly look tired, reporters look inquisitive, mathematicians look studious. Judges become grave, even when off the bench, and the man who has had domestic trouble looks all broken up. An example of the ludicrous side of this subject is to see a third-class lawyer stalking around a police court looking wise as an owl. The business makes the face.

